# Hearing loss and respiratory health symptoms among large-scale sawmill workers of the timber processing factories within the Gert Sibande District Municipality: a comparative cross-sectional study

**DOI:** 10.1186/s12889-023-16086-9

**Published:** 2023-06-21

**Authors:** Moeletsi Rathipe, Selepeng France Raphela

**Affiliations:** 1 Department of Life Sciences, Central University of Technology, Bloemfontein, Free State South Africa; 2 Department of Clinical Sciences, Central University of Technology, Bloemfontein, Free State South Africa

**Keywords:** Wood dust, Respiratory symptoms, Noise, Sawmill workers, Hearing loss

## Abstract

**Background:**

Occupational exposure to wood dust may cause respiratory illnesses, while prolonged exposure to loud noise may cause noise-induced hearing loss.

**Objective:**

The objective of the study was to assess the prevalence of hearing loss and respiratory symptoms among large-scale sawmill workers within the Gert Sibande Municipality in Mpumalanga Province, South Africa.

**Methods:**

A comparative cross-sectional study consisting of 137 exposed and 20 unexposed randomly selected workers was undertaken from January to March 2021. The respondents completed a semi-structured questionnaire on hearing loss and respiratory health symptoms.

**Data analyse:**

The data was analysed using Statistical Package for Social Sciences (SPSS) version 21 (Chicago II, USA). The statistical analysis of the difference between the two proportions was done using an independent student t-test. The level of significance was set at *p* < 0.05.

**Results:**

There was a statistically significant difference between the exposed and unexposed workers on the prevalence of respiratory symptoms like phlegm (51.8 vs. 0.0%) and shortness of breath (chest pain) (48.2 vs. 50%). There was also a statistically significant difference between the exposed and unexposed workers on the signs and symptoms of hearing loss like tinnitus (ringing in the ears) (50 vs. 33.3%), ear infections (21.4 vs. 66.7%), ruptured ear drums (16.7 vs. 0.0%), and ear injuries (11.9 vs. 0.0%). The exposed workers reported always wearing personal protective equipment (PPE) (86.9%) compared to the unexposed workers (75%). The reason for not wearing PPE consistently by the exposed workers was due to not being available (48.5%), compared to the unexposed workers who reported other reasons (100%).

**Conclusion:**

The prevalence of respiratory symptoms among the exposed workers was higher than that of the unexposed workers, except for chest pains (shortness of breath). The prevalence of symptoms of hearing loss among the exposed workers was higher than the unexposed workers, except for ear infections. The results suggest that measures should be implemented at the sawmill to help protect workers’ health.

## Background

Wood dust, endotoxins, and allergenic fungi are the main health hazards encountered in wood processing [1]. Occupational exposure to wood dust and the biological hazards associated with wood dust, endotoxins, (1— > 3)-β-D- glucan, gram-negative bacteria, and fungi has been associated with respiratory symptoms among woodworkers [1, 2]. Respiratory symptoms from exposure to wood dust in the workplace have been investigated in a number of studies [3–22]. These studies produced somewhat contradictory results, depending on the type, extent, and duration of exposure to wood dust in the workplace, the characteristics of the workers studied (age, smoking status, et cetera), the nature of their work activities, as well as the type of study [3]. Ill-health due to the inhalation of wood dust result in the worker being unable to meet the demands of their job, becoming sick more often, being absent more often, and eventually retiring earlier [23]. Workers exposed to wood dust have a higher prevalence of respiratory symptoms than unexposed workers because when symptoms develop during the course of their work, those more severely affected tend to leave or change jobs, leaving the healthier ones behind [24]. Despite the potentially dangerous nature of woodwork, government health departments have paid little attention on respiratory symptoms caused by occupational exposure to wood dust at the large-scale sawmills [23].

Noise-induced hearing loss (NIHL) is also becoming more common in underdeveloped countries, where noise levels are frequently uncontrolled and ear protection is lacking [25–27]. Workers in industries like mining, construction, printing, sawmills, and crushers are particularly at risk [28]. There have been limited studies on hearing loss among workers in these industries [28]. Many studies have shown that occupational exposure to noise cause noise-induced hearing loss [27–36]. Although considerable information is needed to precisely anticipate the future trends of hearing loss caused by noise, ototoxic medication and ear infections that contribute to development of noise-induced hearing loss [34, 37–39]. A lack of national policies that promote access to hearing care, as well as a lack of understanding among policymakers and the general public about hearing loss, its impact, and management have slowed down public health activities in this field [29, 40–42].

Long-term exposure to industrial noise have been shown to increase risk of hearing loss [28, 35, 36]. Little is known about the signs and symptoms of hearing loss in the large-scale sawmill, where excessive noise is more prevalent and control of noise at work is frequently ignored [27]. There have been limited studies on the prevalence of respiratory symptoms caused by exposure to wood dust at the large-scale sawmills. The purpose of the study was to determine the prevalence of hearing loss and respiratory symptoms among large-scale sawmill workers within the Gert Sibande District Municipality in the Mpumalanga Province, South Africa. The findings of this study will provide new knowledge on the signs of hearing loss and respiratory symptoms among sawmills and provide data useful to planning and design of interventions aimed at reducing wood dust and noise in the wood industry.

## Methods

### Setting of the study

The study was conducted at the large-scale sawmill factories located within the Gert Sibande District Municipality in Mpumalanga Province, South Africa. A comparative cross-sectional study consisting of 137 exposed and 20 unexposed workers randomly selected using simple random sampling was undertaken at the large-scale sawmill factories. Furthermore, the study was conducted from January to March 2021. The estimated sample size for the study was 236 based on the Cochran formula for determining sample size with a 95% level of confidence, a 0.5 estimated population proportion, and a 5% margin of error [5, 7, 26]

There were 12 sawmill factories located within the Gert Sibande District municipality. Out of 12 sawmills, two were the largest, while 10 were medium-size sawmills. The study focused on large-scale sawmills because they were more operational than medium- or small-scale sawmills. This criterion was chosen because large sawmills have more workers than medium sawmills, and their operations represent average sawmill enterprises within the Get Sibande District. The two largest sawmills were selected based on their large size, location, and type of wood being processed [1, 42]. The types of trees being processed were predominantly pine, mostly *Pinus radiata*, *Pinus patula*, *Pinus Elliottii*, and others.

### Participants

Sawmill workers were grouped into exposed and unexposed groups. The exposed group comprised of operators, packers, receivers, artisans, stackers, feeders, general workers, and cleaners, while the unexposed group comprised of administration and other office workers whose jobs did not involve wood dust and noise exposure under normal circumstances [1]. The inclusion criteria involved all consenting male and female workers aged between 18 and 65 years who had been in continuous employment for a minimum period of one year and who were willing to participate in the study. Those excluded were workers who did not give consent or were not willing to participate and workers with less than one year of experience at the time of the study.

### Validity and reliability

The questionnaire was developed based on the research tools used in previous studies [3, 7, 8, 16, 18, 20, 23, 24, 43–45]. The question was adopted from previous research and literature to suit the needs of the sawmill industry.

### Piloting of the questionnaire

The questionnaire was piloted on six workers in each mill and workshop. The participants of the pilot study comprised of a stacker, an operator, a general worker, and three office staff. This was done to check for clarity of the questions, content validity, and reliability of the questionnaire. Feedback from the pilot study was incorporated into the final questionnaire. The participants of the pilot study were excluded from the main study. The questionnaire was reviewed by experts in the field of occupational hygiene to determine its applicability to the large-scale sawmill industry. Furthermore, it was pre-tested for content validity with the expectation of obtaining the same responses from all respondents. Closed-ended questions were used to reduce bias and improve the tendency to produce the same results.

### Data collection tool

The data were collected using self-administered questionnaires. The questionnaire was adapted and modified from the British Medical Research Council’s questionnaire on chronic bronchitis and noise exposure [46]. Section A of the questionnaire included questions about participants’ socio-demographic characteristics such as age, gender, education, et cetera. Sections B, C, and D sought to collect data about work-related information such as working hours, job title, work experience, safety training, previous dusty jobs, and health-related information regarding smoking and respiratory symptoms. Section E included questions about the use of personal protective equipment (PPE) [1, 21, 23, 24, 27, 46–58]. The questionnaire was compiled in English and translated into IsiZulu and Afrikaans.

The first step of data collection began with obtaining permission from the Faculty of Health Sciences as well as from the management of the two sawmill factories. Subsequently, meetings were held with participants to explain the aim and objectives of the study, and distribution of information documents and consent forms, and collect the signed consent forms. Next, the questionnaires were administered to sawmill workers. The data were collected with care to prevent any harm by upholding privacy or confidentiality. Moreover, the anonymity of the respondents was guaranteed by omitting their names from the questionnaire. When the respondents did not understand any terminology, the researcher explained using plain language.

The questionnaires were completed during lunch breaks at the sawmills. The participants took about 20 min to complete the questionnaires, and data were collected over five days at each worksite. The researcher collected the completed questionnaires for capture. Thereafter, the questionnaires were cross-checked to ensure that all the necessary information was completed correctly. Finally, data were coded and electronically stored.

### Data analysis

Data were analysed using Statistical Package for Social Sciences (SPSS) version 21 (Chicago II, USA). Categorical responses were tested using the Chi-square test. Fisher's exact test was used when any expected number was less than 5. The statistical analysis of the difference between two proportions was done using an independent student t-test. A significance level of 0.05 was used.

## Results

### The sociodemographic characteristics of the respondents

The study targeted a sample size of 236 respondents and only 157 filled and returned the questionnaires giving a response rate of 67%. Table 1 shows the summary statistics for the demographic characteristics of the respondents. Fifty-three percent (53%) of the exposed group were males while 48% were females and 60% of the unexposed group were males while 40% were females. The mean age (standard deviation) of the exposed was 34,25(23) while unexposed was 5(4,97). The majority of the exposed group (41%) was aged 40 to 49 years while 55% of the unexposed aged 30 to 39 years. More than 92% of the exposed group and 70% of the unexposed were of black ethnicity (*p* < 0.05). The majority of the exposed group (68%) and 75% unexposed were single. Furthermore, 42% of the exposed group had secondary while 50% unexposed had matric. The majority of the unexposed group appears to be more educated had matric and tertiary than exposed who had secondary and matric.

**Table 1:** Demographic characteristics of sawmill workers in timber processing factories, Gert Sibande District, 2022

**Variable**	**Exposed** (*n* = 137)	**Unexposed** (*n* = 20)	***p*-value**
**Gender/sex**	**n (%)**	**n (%)**	
Females	65 (47.45)	8 (40)	0.533^a^
Males	72 (52.55)	12 (60)	
**Age group (years)**	mean(SD) = 34,25(23)	mean(SD) = 5(4,97)	
20 to 29	16 (11.68)	2 (10)	0.047^c^
30 to 39	52 (37.96)	11 (55)	
40 to 49	56 (40.88)	7 (35)	
50 to 59	13 (9.49)	0(0)	
**Ethnicity/race**			
Black	127 (92.70)	14 (70)	< 0.002^a^*
White	10 (7.30)	6 (30)	
**Marital status**			
Single	93 (67.88)	15 (75)	0.817^b^
Married	34 (24.82)	5 (25)	
Divorced	8 (5.84)	0(0)	
Widowed	2 (1.46)	0(0)	
**Level of education**			
None	4 (2.92)	0(0)	0.077^c^
In-house	3 (2.19)	0(0)	
Primary	8 (5.84)	0(0)	
Secondary	58 (42.34)	2(10)	
Matric	47 (34.31)	10(50)	
Tertiary	17 (12.41)	8(40)	

^a^Chi-Square test

^b^Fisher’s exact test

^c^Student t-test

*Significant at *p* < 0.05

### Work-related information of the respondents

The work-related information of the respondents is presented in Table 2. A high proportion of the exposed group (30%) were general workers while 70% of the unexposed group had other job titles. Forty-six percent (46%) of the exposed and 30% unexposed reported having worked in a dusty area before. Ninety-thee percent (93%) of the exposed and 100% of unexposed reported attended safety training. Forty-four percent (44%) of the exposed and 45% unexposed had a working experience of between 3 to 10 years. Fifty-five percent (55%) of the exposed worked for a duration of 9 h a day (45 h a week) while 55% of unexposed work 8 h a day (40 h a week).

**Table 2:** Work-related information of sawmill workers in timber processing factories, Gert Sibande District, 2022

**Variable**	**Exposed** (*n* = 137)	**Unexposed** (*n* = 20)	***p-*value**
**Job title**	**n (%)**	**n (%)**	
Operator	35 (25.55)	0(0)	n/a
Packer	8 (5.84)	0(0)	
Receiver	2 (1.46)	0(0)	
Artisan	4 (2.92)	0(0)	
Stacker	31 (22.63)	0(0)	
Feeder	1 (0.73)	0(0)	
Supervisor	5 (3.65)	0(0)	
General worker	41 (29.93)	0(0)	
Cleaner	2 (1.46)	0(0)	
Other	8 (5.84)	20 (100)	
**Previous dusty jobs**			
Yes	63 (45.99)	6 (30)	< 0.0132^c*^
No	74 (54.01)	14 (70)	
**Received safety training**			
Yes	127 (92.7)	20 (100)	0.363^b^
No	10 (7.3)	0(0)	
**Working experience (years)**			
1 to 2	48 (35.04)	4 (20)	0.058^c^
3 to 10	60 (43.8)	9 (45)	
11 to 20	25 (18.25)	6 (30)	
21 to 30	4 (2.92)	1 (5)	
**Woking hours**			
8 h	59 (43.07)	11 (55)	0.152^c^
9 h	75 (54.74)	9 (45)	
10 h	3 (2.19)	0(0)	

*N/A* Not assessed

^a^Chi-Square test

^b^Fisher’s exact test

^c^Student t-test

^*^Significant at *p* < 0.05

### Prevalence of respiratory symptoms among the respondents

The results in Table 3 show the prevalence of respiratory symptoms and health-related information reported by the respondents. There was a statistical significant difference among the groups for phlegm (51.8% vs. 0.0%) and chest pain/ shortness of breath (48.2% vs. 50%) (*p* < 0.05). But the prevalence of cough (13.1% vs. 10%), nose and throat irritations (8.03% vs. 5%) and chest-related illness (2.9% vs. 0.0%) among exposed were higher than unexposed but didn’t reach statically significant difference between groups. Fifty percent (50%) of coughs among the exposed group were reported to occur more frequently in the morning, 22% during the day and 28% at night while 50% unexposed group reported to occur in the morning and night (*p* < 0.05). In addition, 61% of coughs among the exposed were reported to last between 1 to 3 days, 17% between 3 to 5 days and 22% more than 5 days while 100% of the unexposed group reported to last between 1 to 3 days. Two percent (2%) of the exposed group reported other health-related conditions or diseases comprising of heart failure, chest problems and pneumonia while 1% reported pulmonary tuberculosis and 10% of the unexposed group reported chest-related problems.

**Table 3:** Respiratory symptoms and health-related information of sawmill workers in timber processing factories, Gert Sibande District, 2022

**Variable**	**Exposed** (*n* = 137)	**Unexposed** (*n* = 20)	***p*-value**
**Do you suffer from nose and throat irritations?**	**n (%)**	**n (%)**	
Yes	11 (8.03)	1 (5)	0.421^c^
No	126 (91.97)	19 (95)	
**Do you cough?**			
Yes	18 (13.14)	2 (10)	0.371^c^
No	119 (86.86)	18 (90)	
**When do you cough?**			
Morning	9 (50)	1 (50)	< 0.0270^c*^
During	4 (22.22)	0(0)	
Night	5 (27.78)	1 (50)	
**How long the cough last?**			
1 to 3	11 (61.11)	2 (100)	0.110^c^
3 to 5	3 (16.67)	0(0)	
More than 5 days	4 (22.22)	0(0)	
**Do you produce phlegm?**			
Yes	71 (51.8)	0(0)	< 0.03^c*^
No	66 (48.2)	20 (100)	
**Do you suffer from chest pains/shortness of breath when coughing?**			
Yes	66 (48.18)	10 (50)	< 0.002^c*^
No	71 (51.82)	10 (50)	
**Have you ever suffered from chest related illness in the past?**			
Yes	4 (2.92)	0(0)	0.4052^c^
No	133 (97.08)	20 (100)	
**Have you had similar illness like this in the past?**			
Yes	11 (8.03)	0(0)	0.4217^c^
No	126 (91.97)	20 (100)	
**Do you suffer from one of the following health conditions?**			
Heart failure/problem	3 (2.19)	0(0)	0.3838^c^
Pneumonia	2 (1.46)	0(0)	
Pulmonary tuberculosis	1 (0.73)	0(0)	
Chest related problem	3 (2.19)	2 (10)	
None of the above	128 (93.43)	18 (90)	

^a^Chi-Square test

^b^Fisher’s exact test

^c^Student t-test

^*^Significant at *p* < 0.05

### Smoking status of the respondents

The smoking status and other health-related information of the respondents are shown in Table 4. Eighteen percent (18%) of the exposed group and 45% of the unexposed were smokers and smoke more than 25 packets of cigarettes a week (*p* < 0.05). Sixty-eight percent (68%) of the exposed group and 11% of the unexposed group reported planning to cut down on smoking and 60% exposed and 11% unexposed reported requiring assistance to stop smoking (*p* < 0.05). Eighty-five percent (85%) of exposed and 100% unexposed reported being subjected to medical fitness tests. Sixteen percent (16%) of exposed and 15% of unexposed reported latest medical test reports to require follow-ups on health-related conditions; 55% and 100% respectively. The exposed group appear to have less smokers (18%) than the unexposed (45%). This may be attributed to the 60% of males that were more than 40% of females and may be reluctant to quit smoking due to addiction.

**Table 4:** Smoking status and health-related information of sawmill workers in timber processing factories, Gert Sibande District, 2022

**Variables**	**Exposed** (*n* = 137)	**Unexposed** (*n* = 20)	***p*-value**
**Do you smoke?**	n (%)	n (%)	
Yes	25 (18.25)	9 (45)	< 0.0161^b*^
No	112 (81.75)	11 (55)	
**What type of substance do you smoke?**			
Cigarette	19 (76)	9 (100)	0.478^b^
Dagga	1 (4)	0(0)	
Other	5 (20)	0(0)	
**How many cigarette packets you smoke per week?**			
10 packets	0(0)	0(0)	< 0.0139^b*^
15 packets	3 (12)	0(0)	
20 packets	6 (24)	1 (11.11)	
25 packets	2 (8)	3 (33.33)	
More than 25 packets	14 (56)	5 (55.56)	
**Are you planning to stop smoking?**			
Yes	17 (68)	1 (11.11)	< 0.0056^b*^
No	8 (32)	8 (88.89)	
**Do you need assistance to stop smoking?**			
Yes	15 (60)	1 (11.11)	< 0.009^b*^
No	10 (40)	8 (88.89)	
**Does the company subject you to medical fitness test?**			
Yes	117 (85.4)	20 (100)	0.078^b^
I don't know	20 (14.6)	0(0)	
**Does your medical test report require follow up?**			
Yes	22 (16.06)	3 (15)	< 0.000^b*^
No	31 (22.63)	17 (85)	
Don’t know	84 (61.31)	0(0)	
**Follow up relate to the following**			
Hearing loss	8 (36.36)	0(0)	0.634^b^
Heart disease	2 (9.09)	0(0)	
Other health condition	12 (54.55)	3 (100)	

^a^Chi-Square test

^b^Fisher’s exact test

^c^Student t-test

*Significant at *p* < 0.05

### Utilization of personal protective equipment (PPE) by the respondents

The utilisation of PPE by participants is reflected in Table 5. One-hundred and thirty of exposed workers reported wearing a helmet (95%), 131 reported using ear plugs (96%), 12 reported using safety glass (9%), 16 reported using a respiratory mask (12%), 119 reported using protective gloves(87%), 103 reported wearing overalls (75%), 45 reported using apron(33%), 99 reported wearing safety boots (72%) and five unexposed workers reported wearing a helmet(25%), five reported wearing ear plugs (25%), four reported wearing safety glass(20%), one reported wearing protective gloves (5%) and five reported wearing safety boots(25%). The most frequently worn PPE by the exposed group were ear plugs (96%) and the least worn were respiratory masks (12%) and safety glasses (9%) while the unexposed group was a helmet, ear plugs and safety boots (25%) and least worn PPE being protective gloves (5%). Eighty-seven percent (87%) of the exposed group reported always wearing PPE while 75% of the unexposed group wear it sometimes (*p* < 0.05). The reason for not wearing PPE by the exposed group was due to not being available (48%) while 100% unexposed indicated other reasons (*p* < 0.05). The exposed group appears to use PPE more often than the unexposed due to the fact they are mostly exposed to noise and wood dust than the unexposed group.

**Table 5:** Utilisation of PPE by sawmill workers in timber processing factories, Gert Sibande District, 2022

**Variables**	**Exposed** (*n* = 137)	**Unexposed** (*n* = 20)	***p*-value**
**Type of PPE worn**	n (%)	n (%)	
Helmet	130 (94.89)	5 (25)	1^a^
Ear plugs	131 (95.62)	5 (25)	1^a^
Safety glass	12 (8.76)	4 (20)	1^a^
Respiratory mask	16 (11.68)	0(0)	1^a^
Protective gloves	119 (86.86)	1 (5)	1^a^
Overalls	103 (75.18)	0(0)	1^a^
Apron	45 (32.85)	0(0)	1^a^
Safety boots	99 (72.26)	5 (25)	1^a^
**How often do you wear PPE?**			
Always	119 (86.86)	0(0)	< 0.000^b*^
Most of the times	8 (5.84)	0(0)	
Sometimes	9 (6.57)	15 (75)	
Never	1 (0.73)	5 (25)	
**Why you do not wear protection constantly?**			
Not available	16 (48.48)	0(0)	< 0.006^b*^
Uncomfortable	5 (15.15)	0(0)	
Make difficult breathing	5 (15.15)	0(0)	
Can’t hear properly	4 (12.12)	0(0)	
Other reasons	3 (9.09)	3 (100)	

^a^Chi-Square test

^b^Fisher’s exact test

^c^Student t-test

*Significant at *p* < 0.05

### The prevalence of hearing loss among the participants

The prevalence of hearing loss among participants is shown in Table 6. The most prevalent signs and symptom of hearing loss among exposed was tinnitus (ringing in the ears) (50%), ear infections (21%), rupture ear drums and ear injuries (17%) while unexposed were ringing in the ears (33%) and ear infections (66%) (*p* < 0.05). Four percent (4%) of exposed and 5% unexposed reported having a history of hearing loss. The exposed group reported a higher percentage of sings and symptoms of hearing loss than the unexposed even though 96% of ear plugs were reported to be worn by the exposed than 25% unexposed. The reason for the difference may be attributed by 48% of PPE that was reported not being even though 87% of the exposed reported always wear PPE than 75% of unexposed who reported wear it sometimes.

**Table 6:** Prevalence of hearing loss among sawmill workers in timber processing factories, Gert Sibande District, 2022

**Variables**	**Exposed** (*n* = 42)	**Unexposed** (*n* = 3)	***p*-value**
**Do you experience the following condition or symptoms of hearing loss?**	**n (%)**	**n (%)**	
Tinnitus (ringing in the ear)	21 (50)	1 (33.33)	< 0.036^c*^
Ear infection	9 (21.43)	2 (66.67)	
Rupture ear drum	7 (16.67)	0(0)	
Ear injury	5 (11.9)	0(0)	
Other, specify	0(0)	0(0)	
**Do you have previous history of hearing loss?**			
Yes	6 (4.38)	1 (4.46)	1^b^
No	131 (95.62)	19 (95.54)	

^a^Chi-Square test

^b^Fisher’s exact test

^c^Student t-test

^*^Significant at *p* < 0.05

## Discussions

We found a higher prevalence of respiratory symptoms in the exposed workers compared to the unexposed workers, with a statistically significant difference for phlegm and shortness of breath (chest pains). The prevalence of cough (13.1% vs. 10%), nose and throat irritations (8.0% vs. 5%), and chest-related illness (2.9% vs. 0.0%) was higher in the exposed workers compared with the unexposed workers, with no statistically significant difference between the groups. These results vary with those of previous studies [3, 8, 13, 16, 23, 59–67]. The prevalence of phlegm (51.8%) in the exposed workers was higher than in earlier studies [3, 16, 17, 19, 20, 22, 23, 43, 65, 68–70]. The reasons for the difference might be due to poor practice on the use of respiratory protection equipment (RPE). Working without using RPE increases the risk of respiratory symptoms [1, 11, 21, 23, 57, 58, 71, 72]. Likewise, the prevalence of shortness of breath (chest pains) in the unexposed workers (50%) was higher compared to the exposed workers (48.2%). However, previous studies reported a higher prevalence of respiratory symptoms in exposed workers than unexposed workers [3, 15, 16, 19, 20, 23, 65, 69, 70]. The difference might be due to the smaller sample size used in the present study, which could increase the possibility of type 2 error in the analysis. This could be further explained by the study setting and air pollution at the sawmills.

The frequency of cough in the morning (50%) and during the day (22.2%) was greater in the exposed workers compared with the unexposed workers, with the exception of cough at night (50%). The findings varied with those of previous studies [18, 44, 68]. Eighteen percent (18%) of those exposed and 45% of those unexposed were smokers who smoked more than 25 packets of cigarettes a week. Precious studies showed that those smoking cigarettes were more likely to have coughs, phlegm, and wheezing [21]. Likewise, smoking is a predictor of hearing loss and respiratory symptoms [12, 21, 23, 71]. The previous studies found no association between smoking and the development of noise-induced hearing loss [27, 31]. This study found that signs and symptoms of hearing loss were higher in the exposed workers than the unexposed workers, with statistically significant differences for tinnitus (ringing in the ears), ruptured ear drums, and ear injuries, although ear infections (21% vs. 66.7%) were lower in the exposed workers than the unexposed workers. These findings were inconsistent with earlier studies [26, 73, 74]. Tinnitus could have occurred due to a previous history of work-related exposure to loud noise [75, 76] while an ear infection could have resulted after an ear injury leading to pus discharge in the ear [77–80]. However, other risk factors for hearing loss include age, family history, ear infections, exposure to ototoxic medications like aminoglycosides and nicotine, and exposure to loud sound [71, 81–89].

The employment duration of the exposed and unexposed workers was between 3 and 10 years. Workers with 10 years of employment in dusty jobs was reported to have a higher prevalence of respiratory symptoms than the unexposed workers [3, 15, 23] because those who are worse affected tend to leave or change jobs, leaving the healthier ones behind [23, 24]. Fifty-five percent of the exposed and unexposed workers worked 9 and 8-h shifts in a day. Sawmill workers and controls were reported to work long hours in a day to maximize production [73, 90]. Working for more than 9 h without hearing protection was reported to increase the risk of hearing loss [27, 33, 49, 73, 73, 74]. Past occupational dust exposure was reported to be a major factor in the development of chronic respiratory symptoms [15, 27, 32]. Likewise, past history of exposure to occupational noise was reported to be a major factor in the development of tinnitus [75]. However, poor practise on the use of hearing protective devise (HPD) properly and consistently is linked with hearing loss among workers.

The least worn personal protective equipment (PPE) among the exposed workers were respiratory masks (12%) and safety glasses (9%). This results were consistent with previous studies [15, 21, 55]. Poor practice in the use of respiratory protection equipment (RPE) was associated with higher prevalence of respiratory symptoms among wood workers [36, 58, 71, 72]. Employees who wear RPE properly and consistently can have lower respiratory symptoms [55, 56, 91]. The reason for not wearing PPE was reported as not being available (48%) by the exposed workers, while 100% of the unexposed indicated other reasons. This was consistent with previous studies [73, 74, 92–98]. Employers do not provide appropriate PPE to workers. The exposed workers (92.7%) and the unexposed workers (100%) reported attending occupational health and safety training at the sawmill. This was inconsistent with previous studies that reported none of the workers attended safety training [55, 78]. Furthermore, 42% of the exposed group in the present study had secondary education, while 50% of the unexposed had matric. The level of education for the exposed worker below secondary may display ignorance on the use of PPE, although the high percentage were those using ear plugs (95.6%) than RPE (11.7%), which was consistent with previous studies [15, 21, 55, 97, 99].

### Limitations of the study

One of the limitation for this study is the healthy worker effect, as the workers who developed the symptoms may have quit the job and other moved to a less dusty job. The use of a self-administered questionnaire in this study can lead to an overestimation of the problem. Using a self-administered questionnaire may cause participants to recall bias. Besides, since the study was cross-sectional, it will not show the cause-effect relationship of the problem. Assessment of respiratory symptoms and hearing loss was made based on self-report and was not validated using medical records. The sample size for the unexposed was relatively small, which could have certain implications for the data interpretation. The study did not investigate the possibility of ototoxicity in the participated industries.

## Conclusion

We found a higher prevalence of respiratory symptoms in the exposed workers compared to the unexposed workers, with a statistically significant difference for phlegm and shortness of breath. The study further establish signs and symptoms of hearing loss in the exposed workers compared to the unexposed workers, with statistically significant difference for tinnitus (ringing in the ears), ear infections, ruptured ear drums and ear injuries. The results have strong implications on government, relevant agencies, policymakers, employers, employees, and representatives for enforcement of stricter legislation to limit exposure, advocacy on hearing loss and respiratory symptoms, and its impact on the workers. Workers smoking cigarettes or tobacco to be encouraged to stop smoking because smoking may increase the risks of respiratory symptoms or development of high or low frequency hearing loss when associated with exposure to wood dust and noise.

## Data Availability

The datasets used and/or analysed during the current study are available from the corresponding author on reasonable request.

## Abbreviations

*et cetera*: And other similar things
HPD: Hearing Protective Device
PPE: Personal Protective Equipment
RPE/D: Respiratory Protection Equipment/device
NIHL: Noise-Induced Hearing Loss
SPSS: Statistical Package for Social Sciences
